# Palliative care for patients with hematologic malignancies in Germany: a nationwide survey on everyday practice and influencing factors from the perspective of treating physicians

**DOI:** 10.1007/s00277-024-05726-8

**Published:** 2024-03-28

**Authors:** Cordula Gebel, Isabel Kruschel, Steffi Bodinger, Steffen T. Simon, Dennis A. Eichenauer, Anne Pralong, Ulrich Wedding

**Affiliations:** 1 Department of Palliative Care, University Hospital Jena, Friedrich-Schiller University, Jena, Germany; 2 Department of Palliative Medicine, Faculty of Medicine and Cologne University Hospital, Center for Integrated Oncology Aachen Bonn Dusseldorf Cologne, Cologne, Germany; 3 Comprehensive Cancer Center Central Germany (CCCG), Cologne, Germany; 4 First Department of Internal Medicine, Center for Integrated Oncology Aachen Bonn Dusseldorf Cologne, University of Cologne, Cologne, Germany

**Keywords:** Hematologic malignancies, Palliative care, Referral, Integration, Survey, Physicians

## Abstract

Integrating palliative care into the treatment of patients with advanced hematological malignancies (HM) remains challenging. To explore treating physicians’ perspectives on current palliative care practice and to evaluate factors influencing integration, we conducted a nationwide online survey. Based on literature and expert review, the survey addressed the importance of palliative care, communication about life-threatening conditions, challenges in establishing goals of care, and factors influencing the integration of palliative care. 207 physicians treating patients with HM in Germany participated. We used standard descriptive statistics to analyze quantitative data and a content structuring approach. Most physicians considered palliative care in HM to be very important (60.6%) and discussed life-threatening conditions with more than half of their patients (52%), especially when goals of care were changed (87.0%) or when patients raised the topic (84.0%). Disease-related factors, different professional perspectives on prognosis, and patient hopes were the main barriers to changing goals of care, but collaboration with colleagues and multidisciplinary teams provided important support. Time constraints were identified as the main barrier to integrating palliative care. The majority worked well with palliative care teams. Referral processes and conditions were perceived as minor barriers. The study highlights the need to address barriers to integrating palliative care into the management of patients with advanced HM. Future research should aim at optimizing palliative care for patients with HM.

**Supplementary Information:**

The online version contains supplementary material available at 10.1007/s00277-024-05726-8.

## Introduction

Physicians play a key role in integrating palliative care into the patients’ management as they identify patients who may benefit from palliative care and initiate and coordinate appropriate treatment in most cases [1]. Palliative care offers significant opportunities to improve quality of life, manage physical and psychological symptoms, and formulate care plans. It appears to be particularly effective when implemented early and seamlessly integrated into treatment [2, 3].

In contrast to patients with solid tumors, patients with hematologic malignancies (HM) more often receive intensive care, are more frequently hospitalized [4–6], and are more commonly treated with aggressive therapies at the end of life. Consequently, they more often die in the hospital [4, 5, 7–9]). Despite a comparable symptom burden, patients with HM are less likely to receive palliative care, often only when they are close to death [8, 10–14]. However, these patients could benefit from earlier integration of palliative care, as suggested by several studies [12].

The reasons for the late integration of palliative care in patients with HM are complex. LeBlanc and colleagues have identified and summarized several barriers. First, palliative care is often equated with end-of-life care. Second, the uncertain prognosis of many HM and a wider range of therapeutic options lead to uncertainties in the transition between curative and palliative care. Third, structural challenges related to the availability of palliative care services and reimbursement are barriers [15, 16]. In addition, one study found significant differences in attitudes toward end-of-life care between hematologists and oncologists. Hematologists were more likely to report a sense of failure if they could not change the course of the disease and were less comfortable discussing death and dying and referring patients to hospice care [10]. In a qualitative study by McCaughan and colleagues, it was noted that the close relationship between patients and the hematology team may delay palliative care discussions and palliative care team involvement [17, 18]. However, patients expect their treating physicians to initiate the palliative care discussion in many cases [19].

To our knowledge, there are currently no analyses on palliative care for patients with hematological malignancies from the perspective of treating physicians in Germany. Previous research on this topic has focused primarily on Canada [20], the United States [10, 16, 21, 22], and the United Kingdom [17].

### Aim and research question

The main objective of this study is to comprehensively describe the current palliative care of patients with HM from the perspective of treating physicians and to investigate factors that influence the integration of palliative care into the management of this patient group.

The study focuses on the following questions:

1. How is current palliative care for patients with HM perceived by treating physicians in routine practice?
  What is and should be the role of palliative care?
  How is communication about the threat to life and fear of dying implemented in practice?
  What are the challenges in determining when to change goals of care and what support services could facilitate decision making?
2. How do treating physicians evaluate factors that influence the decision to integrate palliative care into the care of patients with HM?

## Materials and methods

### Survey setting and sample

This study is based on a prospective, cross-sectional, nationwide online survey among physicians who treat patients with HM. Physicians may work in both outpatient and inpatient settings. The sample includes experienced specialists in internal medicine and hematology/oncology as well as physicians in training. Cases were excluded if they work exclusively in palliative care or if they only completed the demographic information in the questionnaire and not the content. The results of this study are presented according to the “Checklist for Reporting Results of Internet E-Surveys” [23] (CHERRIES - see Supplementary Table S1).

### Survey instrument

The development of the questionnaire was based on a comprehensive analysis of relevant literature, comparable surveys, and previously published survey instruments [1, 10, 16, 20, 22, 24–28]. A multidisciplinary review of the questionnaire was then conducted by experts (palliative care physicians, general practitioners, hematologists/oncologists, and psycho-oncologists). This review included both methodological and content aspects, assessing appropriateness, feasibility, and face validity.

The pretest was administered to eight physicians who treat patients with HM. This was done to ensure comprehensibility, acceptability, and a balanced distribution of responses. As a result, minor adjustments were made to the questionnaire and five questions were eliminated. After these refinements, the questionnaire was considered to be effective and feasible.

The four categories of the questionnaire that were analyzed are presented in the following. For each category, there was an opportunity to provide information in free text fields. Supplementary Table S2 lists the questions analyzed and their answer options.

1. Demographics and palliative care in routine practice (9 questions): This category includes items on gender, age, specialist status, primary workplace, qualification in palliative care, work experience in the treatment of patients with HM, the proportion of patients with HM among all patients, importance of palliative care, and frequency of palliative care need in daily practice. The answers could be given in a categorical answer format.
2. Talking about the threat to life and the fear of dying (2 questions): The first question asks the respondent to estimate the percentage of patients with HM with whom the topic of “threat to life /fear of dying” is discussed. Answers are given in a categorical format. The second question asks about the typical time when this topic is discussed. Multiple answers are possible.
3. Change in goals of care (2 questions): Change in goals of care represents a shift from the goal of cure to limiting selected life-prolonging measures (e.g. cardiopulmonary resuscitation, invasive or non-invasive ventilation) or even limiting therapeutic measures to a purely supportive approach (palliative or allowing the natural course) to optimize quality of life. The questions were taken from the research project “Palliative-supportive therapy offer in allogeneic stem cell transplantation (allo-PaS)” [29]. The first question focuses on the challenges that are perceived in determining the timing of a change in the goals of care. The second question asks about the services that are considered helpful or desirable in the context of clinical decision-making regarding a change in goals of care and discussions with patients and their families.
4. Factors influencing palliative care integration (18 questions): The questionnaire used here was developed by the Palliative Care Early and Systematic (PaCES) working group [1, 20, 27] and systematically translated into German (CG and IK), taking into account cultural and disease-specific circumstances. The PaCES working group uses the Behavior Change Wheel (COM-B) [30] as an evidence-based theoretical model for diagnosing behavioral factors in the integration of specialized palliative care [1, 20]. The COM-B model encompasses the three levels of capability, motivation, and opportunity that determine whether a particular behavior is manifested. The use of this framework allows for a methodical and targeted assessment of influencing factors and barriers, with three specific areas mapped: “Addressing palliative care needs myself”, “Referral to palliative care “, and “Working with palliative care teams”. The answer format was a five-point Likert scale from “strongly disagree” to “strongly agree”.

### Survey process and recruitment

The survey period was from November 10 to December 19, 2023. To identify potential participants, namely physicians who treat patients with HM, a database was created based on publicly available membership lists of the German Professional Association of Hematologists and Oncologists in Private Practice (BNHO) and the German Society of Hematology and Medical Oncology (DGHO). The selection was based on an Internet search of the physicians’ areas of practice, excluding pure research centers, special centers (e.g., breast cancer, sarcoma), pharmacies, and physicians working exclusively with children. During the search, e-mail addresses were added, and in the case of group practices, the general e-mail address was used. The database included a total of 1.493 individuals and 94 group practices.

Recruitment was a two-step process. First, information about the study was provided in the DGHO newsletter and participation in the online survey was requested. In the second step, individuals and group practices identified in the database were contacted twice by a personal e-mail. After two weeks, a reminder was sent. As an incentive to participate, participants were offered the chance to win one of ten vouchers worth €50 each. The online survey was conducted using *LimeSurvey.*

### Data analysis

Statistical analysis was performed with R (version 4.2.1). Quantitative data analysis includes standard descriptive statistics, with mean, standard deviation, percentages, and absolute frequencies.

The answer options for the " Change in goals of care” section were grouped into three categories for each question (question 1: disease-related, physician- and team-related, and patient-related factors; question 2: clinical parameters and guidelines, involvement or collaboration with colleagues or the multidisciplinary team, guidance and training). Percentages were calculated based on the total number of valid cases for each question, both for the answer options and for the categories.

In the section “Factors influencing the integration of palliative care”, the strength of the influencing factors was determined by the calculation of the mean of the responses. Five items had a positive wording, meaning that high scores on these items indicate high capability. Conversely, high scores on other items indicate barriers to integration. Free text responses, open-ended questions, and comments were qualitatively analyzed using the content-structuring approach according to Kuckartz (2018) [31]. The results were inductively summarized into categories and agreed upon by the research team (CG and SB).

## Results

After the invitation to participate was published in the newsletter of the DGHO, the survey was sent to a total of 1587 e-mail addresses. Of these, 112 were undeliverable. A total of 237 persons opened the questionnaire, resulting in a response rate of 16.1%. 21 persons were excluded because they only completed demographic data, and another 9 persons were excluded because they worked in palliative care and therefore had a potential conflict of interest. This left 207 questionnaires for analysis (87.3% completion rate). Of the participants, *n* = 65 (31.6%) used the free text option. In the following sections, the qualitative results are presented in addition to the respective topic. A summary table of the qualitative responses and categories can be found in Supplementary Table S3.

### Characteristics of respondents

Respondent characteristics are shown in Table 1. The majority of respondents were male (57.1%). The age distribution showed a wide range, the majority was between 40 and 59 years of age. Regarding specialist status, 87.6% of respondents were specialists in hematology and oncology, while 12.4% were not board-certified. The majority of participants, 64.2%, were employed in hospitals. 56.1% of participants reported more than 15 years of experience in hematology.

### Palliative care in routine practice

53.6% of respondents reported having additional training in palliative care. Regarding the proportion of patients with HM, the majority of respondents (29.8%) indicated that 26–50% of their patients belonged to this group. The frequency of need for palliative care varied, with 48.2% reporting that this need was frequent in their daily practice.

The importance of palliative care for patients with HM was rated as high by 60.6% of respondents, while 32.6% rated it as moderate.

**Table 1:** Demographics and palliative care in everyday life

Characteristic	Answer options	n(%)
Sex	Female	87 (42.9%)
	Male	116 (57.1%)
	Missing	4
Age (years)	<40	49 (24.0%)
	40–49	66 (32.4%)
	50–59	65 (31.9%)
	>60	24 (11.8%)
	Missing	3
Specialist status	Specialist in hematology and oncology	177 (87.6%)
	Assistant physician	25 (12.4%)
	Missing	5
Primary workplace	Practice	73 (35.8%)
	Hospital	131 (64.2%)
	Missing	3
Work experience in the treatment of patients with hematologic malignancies (years)	<2	3 (1.5%)
	2–5	21 (10.7%)
	6–10	26 (13.3%)
	11–15	36 (18.4%)
	>15	110 (56.1%)
	Missing	11
Additional training in palliative care (“board certification palliative care”)		111 (53.6%)
Proportion of patients with hematological malignancies out of all patients	<5%	8 (3.9%)
	6–25%	47 (22.9%)
	26–50%	61 (29.8%)
	51–75%	28 (13.7%)
	76–95%	37 (18.0%)
	>95%	24 (11.7%)
	Missing	2
Frequency of need for palliative care in patients with hematologic malignancies in daily practice	Very Rarely	5 (2.6%)
	Rarely	14 (7.3%)
	Occasionally	57 (29.5%)
	Frequently	93 (48.2%)
	Very Frequently	24 (12.4%)
	Missing	14
The importance of palliative care for patients with hematologic malignancies in medical practice	High importance	117 (60.6%)
	Medium importance	63 (32.6%)
	Peripheral importance	13 (6.7%)
	Missing	14

*Note* Relative percentage, without missing values

### Talking about the threat to life and the fear of dying

Table 2 provides insight into communication practices regarding the topic of “threat to life /fear of dying” in patients with HM. The percentage distribution showed a wide variation, ranging from less than 5% to more than 95%, in the frequency this topic was discussed. 48% of respondents reported discussing this topic with less than half of their patients with HM, while 52% reported discussing it with more than half of their patients.

The timing of these discussions was also examined and is reported in Table 2.

Qualitative free-text analyses (Supplementary Table S3) extend the findings by emphasizing that the timing and frequency of discussion of “threat to life /fear of dying” is highly dependent on the specific entity and prognosis of HM. The timing of such discussions may also depend on specific triggers, such as before allogeneic stem cell transplantation. In addition, it is suggested that the optimal timing should be determined by the psychological readiness of the patient. Early, open, repeated, and situational discussions are recommended, which should not take place in isolation but as part of a broader conversation.

**Table 2:** Talking about the threat to life and the fear of dying

Question	Answer options	n (%)
With what percentage of your patients with hematologic malignancies do you discuss the topic of “threat to life / fear of dying”?	< 5%	5 (2.5%)
	6–25%	44 (22.0%)
	26–50%	47 (23.5%)
	51–75%	41 (20.5%)
	76–95%	39 (19.5%)
	> 95%	24 (12.0%)
	Missing	7
With your patients with hematologic malignancies, at what point do you typically bring up the topic of “threat to life /fear of dying”? (multiple answers possible)	When goals of care change	174 (87.0%)
	Whenever patients/relatives raise the issue themselves	168 (84.0%)
	When cancer is diagnosed	154 (77.0%)
	In case of acute deterioration	153 (76.5%)
	In the pre-terminal/terminal phase	123 (61.5%)
	At the start of treatment	69 (34.5%)
	During a stable phase	22 (11.0%)
	None of the above / unable to answer	1 (0.5%)
	Missing	7

*Note* Relative percentages, without missing values. Multiple answers sorted by case size in descending order

### Change in goals of care: challenges and helpful resources

Table 3 provides an overview of the challenges encountered in determining when to change the goals of care for patients with HM. Disease-related factors were the most frequently cited challenges (90.1%). The availability of tumor-directed treatments, even if less effective, was the most frequently cited barrier in this category (63.5%). Physician- and team-related factors were cited as challenges by 85.4% of participants, particularly differing professional assessments of prognosis or patient preferences. Patients’ hope for improvement with additional therapies was cited as a challenge by more than half of respondents (59.4%). In the qualitative analysis (see Supplementary Table S3), other challenges were identified, such as different patient and family perspectives and specific patient cohorts, such as young patients.

Table 3 also illustrates what physicians found helpful in making decisions about changes in goals of care and discussions with patients and their families. Involvement or collaboration with colleagues or the multidisciplinary team was the most frequently cited category (85.4%), followed by clinical parameters and guidelines (46.1%), and guidance and training (36.3%). The qualitative analysis revealed that collegial exchange and team consultation can be both a challenge and a resource. For example, a specialist in hematology and oncology emphasized that tumor conferences can be challenging because they sometimes discuss treatment options that are feasible according to guidelines but not in reality (ID146). In addition, multiprofessional discussions can be an important resource because the professionals involved are more likely to support the decisions made (ID177). The importance of observing discussions about changing goals of care or receiving guidance from experienced colleagues is relatively low (15.0% and 15.5%, respectively). Qualitative analysis suggests that this may be because not all experienced colleagues can provide effective structured guidance.

The qualitative analysis also emphasizes that discussions about changes in goals of care should primarily be led by the treating physicians and not delegated to other professionals.

**Table 3:** Changing goals of care: challenges and helpful resources

Question	Category /Answer options	n(%)
From a medical perspective, what makes it challenging for you to determine when to change goals of care in patients with hematologic malignancies? (*n* = 192)	**Disease-related factors**	**173 (90.1%)**
	• Having other treatment options available (63.5%) • Long, complicated courses after allogen stem cell therapy (50.5%) • Progression with rapid clinical deterioration (45.8%) • Decision on the limitation of certain therapies (30.2%) • Lack of objective parameters (25.5%)	
	**Physician and team-related factors**	**164 (85.4%)**
	• Prognosis was judged differently by different professional groups (57.3%) • Professional groups have different perceptions of the patient’s wishes for a change in goals of care (56.3%) • Seeing a change in goals of care as a failure of my professional role (5.7%)	
	**Patients-related factor -** Patients hope	**114 (59.4%)**
Which services are or would be helpful to you in making clinical decisions about changes in goals of care and in discussing these decisions with patients and their families? (*n* = 193)	**Clinical parameters and guidelines**	**89 (46.1%)**
	• Have standardized clinical parameters that indicate non-curability (red flags)(37.8%) • Guidelines with key medical criteria and support for conducting and implementing discussions (21.8%)	
	**Involvement or exchange with colleagues or the multidisciplinary team**	**169 (87.6%)**
	• Board on bone marrow transplantation tumor boards, and collaborative decision-making with other colleagues (56.0%) • Involving psycho-oncologists (53.9%) • Involving palliative care physicians in the decision-making process (46.1%) • Involving palliative care physicians in discussions with patients/families (46.1%) • Ethical case discussion (39.9%)	
	**Guidance and Training**	**70 (36.3%)**
	• Training on breaking bad news (23.8%) • Observation of discussions by (experienced) colleagues (15.5%) • Guidance in discussions by (experienced) colleagues (15.0%)	

*Note* Multiple responses possible. All percentages refer to the number of valid cases (*n* = 192/*n* = 193) of the respective question, sorted by case size in descending order in the corresponding category. Formulations have been abbreviated; the exact formulations of the elements are included in Supplementary Table S2

### Factors influencing palliative care integration

**Fig. 1:**
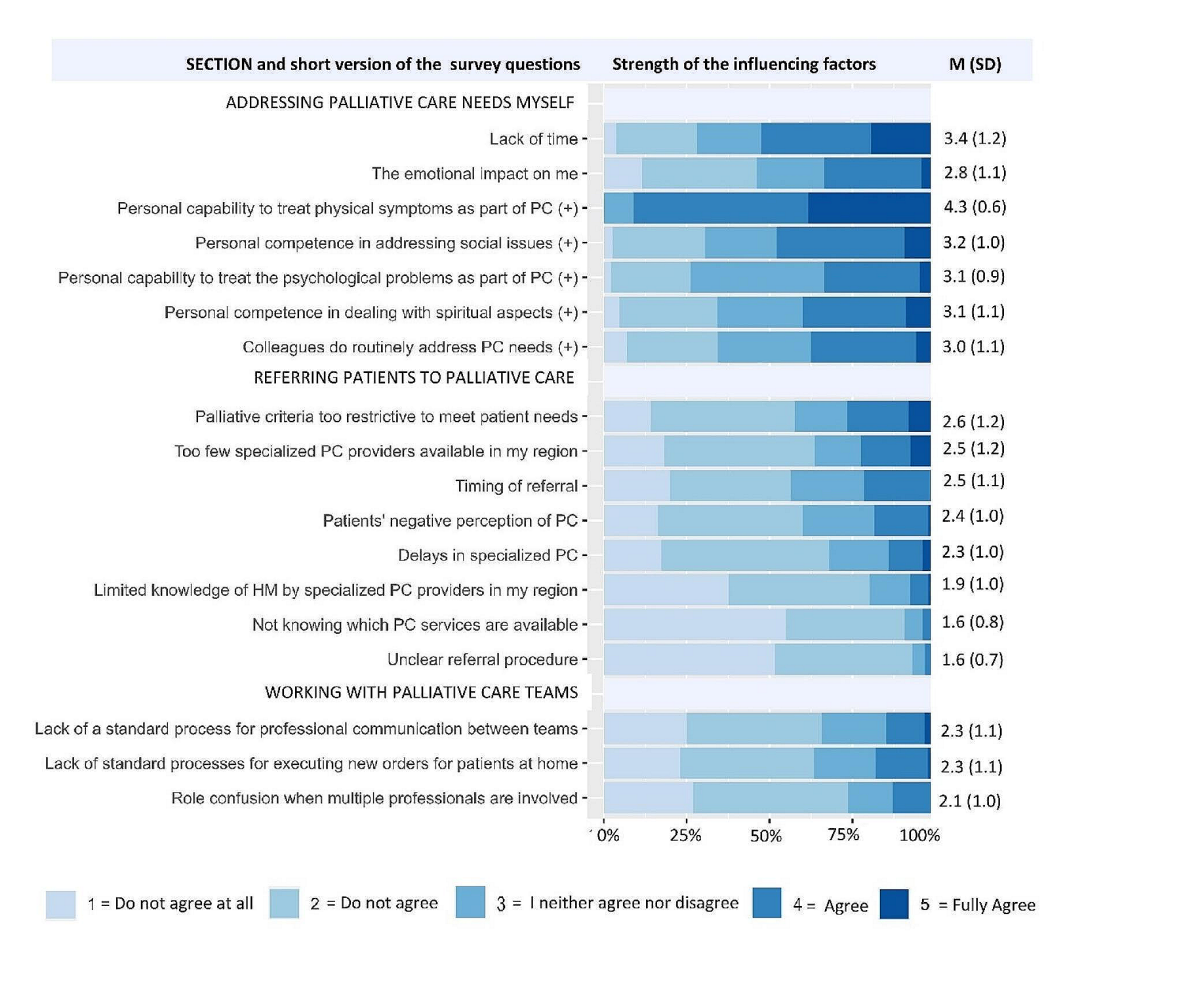
Factors influencing palliative care integration. The strength of the influencing factors was calculated as the mean of the responses. PC = palliative care. (+) = indicates items with positive wording. High scores here indicate high capability. High scores in other cases indicate barriers. The items were sorted in descending order of size in the respective categories. In the case of addressing palliative care needs myself, the items were also sorted according to positively and negatively worded statements

Figure 1 shows the ratings of factors influencing the integration of palliative care in patients with HM. Lack of time (M = 3.4) was the most frequently rated barrier to integration. In the domain “Addressing palliative care by myself”, the remaining items were positively formulated and indicated a high level of competence rather than barriers. In particular, the capability to deal with the patient’s physical symptoms (e.g., pain, shortness of breath) was rated particularly highly (M = 4.3). Other aspects such as dealing with psychological symptoms (M = 3.1), spiritual considerations (M = 3.2), and social issues (M = 3.1) were rated lower. These findings are also reflected in the qualitative analysis, where the importance of clear delineation of responsibilities was emphasized. An expert in hematology and oncology pointed out that medical issues are dealt with either by him or by specialist departments, while other issues are referred to specialist services such as social services, psycho-oncology or hospital chaplains (ID185). When examining contextual factors regarding whether colleagues incorporate palliative care into their routine treatment, a moderate manifestation is observed (M = 3.0). The qualitative analysis complements this by highlighting the importance of robust networks and clinical structures for the integration of palliative care.

In the areas of referral and working with palliative care teams, all mean values were below 3, indicating low to moderate barriers. The barrier with the highest score (M = 2.6) is the perception that the criteria for palliative care are considered too restrictive. Again, 57.0% of respondents disagreed with this statement, while 26.2% agreed. The second most frequently indicated barrier (M = 2.5), the perception that there are too few palliative care providers, was also more frequently disagreed with (63.0%) than agreed with (22.5%). The qualitative analysis indicated occasional capacity bottlenecks in specialized palliative care teams. The vast majority of participants were informed about palliative care services available in the region (M = 1.6) and were familiar with the referral process (M = 1.6). Although lack of awareness among palliative care teams regarding HM and progression was identified as a barrier in the qualitative analysis, it was rated as low overall (M = 1.9).

In the area of working with palliative care teams, it was found that the majority of physicians collaborated well with palliative care services, as emphasized in the qualitative analysis. The qualitative analysis also highlights the importance of the physician’s attitude, both in terms of communication and inclusion of palliative care in treatment.

## Discussion

The majority of physicians surveyed emphasize the importance of palliative care for patients with HM and regularly provide palliative care to their patients. More than half reported that they discuss life-threatening and dying fears with more than 50% of their patients, especially when goals of care changed or when patients or their families initiate the conversation. Disease-related factors posed significant challenges to changing goals of care, while communication with colleagues or multidisciplinary teams provided critical support. Time constraints emerged as the main barrier to palliative care integration. Most physicians experienced minimal barriers to referral and collaboration with palliative care teams.

The results of the study should be considered in the context that more than half of the participants have specific additional training in palliative care. This may have influenced the results by underrepresenting important challenges in integrating palliative care into hematology care.

In our study, 52% of physicians discussed the life-threatening nature of the disease and fear of dying with over half of their patients, while only 12% did so with more than 95% of their patients. Comparatively, another study found that 60.3% of hematologists had prognostic discussions with over 95% of their patients [22]. However, to contextualize the results, it should be noted that the present study does not only include prognostic discussions but also discussions about the emotional component of life-threatening illness and fear of death [22]. The difficulty of including more emotional topics has also been reported in other studies of oncologists [10, 22, 32]. In the field of oncology care, dealing with end-of-life issues is considered one of the most stressful and challenging tasks [33]. Nevertheless, most patients with advanced cancer desire open and early communication about prognosis and end-of-life issues [34].

The vast majority of respondents generally address the issue of threat to life and fear of dying when goals of care change or when the patient asks about it. However, it is not necessarily a single point in time that is relevant, but rather that the topic should be addressed repeatedly, early, and in a manner appropriate to the situation. This finding is also emphasized in other studies, as prognosis and treatment approaches can change the course of disease and patients are receptive to different approaches [22, 34, 35].

The present study confirms the finding reported in other publications that disease-specific factors pose significant challenges when discussing a change in goals of care [6, 35–38] and identifies lack of time as the primary barrier to integrating palliative care for patients with HM [1, 20, 27, 39]. In addition, as other studies have emphasized, patients’ hope is a substantial challenge for treating physicians [35, 39, 40]. This finding is noteworthy because a growing body of research suggests that there is no harm in openly discussing prognosis and progression in the physician-patient relationship [35, 41].

Collaborating with multiprofessional teams and colleagues can be challenging, but it’s essential for integrating palliative care [42, 43]. However, previous studies have shown that oncology, palliative care, and other supportive care teams often work separately [44]. There is also potential for the development of defined roles and tasks in supportive areas, particularly with regard to palliative care, psycho-oncology, social services, and spiritual care. In addition, standardized referral criteria or increased mention of palliative care in treatment guidelines could lead to more frequent and timely referral [45, 46]. Interestingly, this study highlights a lower emphasis on observing conversations about changing goals of care or guidance from experienced colleagues, possibly due to varying abilities to provide structured guidance. Systematic training for physicians and supervisors could improve palliative care integration and decision-making about goals of care through workshops, practical training, and supervision [25, 27].

In this study, physicians rated their ability to manage palliative physical symptoms, such as pain and breathlessness, as good. In contrast, skills in dealing with psychological, spiritual, and social aspects were rated lower. This is consistent with the results of a study of radiation oncologists, who emphasized their confidence in assessing and managing pain and gastrointestinal symptoms, but were less confident in managing anorexia, anxiety, and depression [47]. However, skills in these areas are critical to identifying patients’ needs. One possible support could be the introduction of routine screening to assess patients’ psychological, spiritual, social, and palliative needs. This could enable the effective integration of different support professions [48, 49].

Further barriers to the integration of palliative care have been identified in other studies. These include patients’ negative perceptions of palliative care [1, 20, 39], palliative care teams’ lack of knowledge of HM [28, 50], and referral criteria perceived as too restrictive [20]. All of these criteria were rated as low to moderate barriers by participants in this study. Of particular interest is the discrepancy regarding the limited availability of palliative care services [20, 27, 39]. In this study, most participants reported good knowledge of existing palliative care services, familiarity with the referral process, and effective collaboration with palliative care services. These differences in perceptions may be due to systemic differences in the healthcare system. In Germany, physicians are specialized in hematology and oncology, which means that many physicians who treat patients with HM also treat patients with solid tumors. This could have resulted in a better knowledge of referral pathways and more efficient collaboration with specialist palliative care teams. This association was also found in a survey of hospice care for patients with blood cancers. Physicians who frequently treated patients with solid tumors were more likely to believe that hospice care is beneficial for patients with blood cancers [50].

### Strength and limitations

This study on palliative care in patients with HM is the first nationwide survey from the perspective of physicians treating patients with HM in Germany. It comprehensively examines current palliative care practices, communication, changes in goals of care, and evaluates factors influencing the integration of palliative care.

The present study has several limitations. The sample response rate is an estimate because the total number of physicians treating patients with HM is not known. If we refer only to the database with the email addresses, we have a low response rate compared to other studies (Ranging from 16% to 56%) [20, 28, 47, 51–54]. Nevertheless, the absolute number of participants, 207 physicians who treat patients with HM, is substantial compared to other studies (Ranging from 23 to 349) [10, 16, 20, 22, 50]. However, the response rate limits the representativeness of our results. Qualitative information was provided by 32% of the respondents and can therefore only be used as an indication for explanations. As mentioned above, more than half of the participants had received training in palliative care, so there may be a selection bias. Therefore, our results are likely to underestimate negative perceptions and attitudes toward palliative care. In addition, physician opinions and beliefs may not accurately reflect the circumstances of referral.

## Conclusions

The study results provide a comprehensive insight into the palliative care practices of physicians treating patients with HM in Germany. Palliative care is highly valued and often integrated into treatment. Repeated communication about the threat to life and the fear of dying is considered particularly important. Disease-specific barriers in the context of HM represent a potential challenge for the integration of palliative care, with lack of time being identified as the greatest barrier. Collegial and multiprofessional exchange is highlighted as an important resource. Future research should explore practice-based models of palliative care integration to improve communication and collaboration.

### Electronic supplementary material

Below is the link to the electronic supplementary material.

Supplementary Material 1: Online survey conducted and evaluated according to the CHERRIES checklist

Supplementary Material 2: Analyzed Questions

Supplementary Material 3: Themes from qualitative data

## Data Availability

The data that support the findings of this study are available from the corresponding author upon reasonable request.

## Declarations

The study was conducted in accordance with the Declaration of Helsinki, and approved by the local ethics committee from Jena University Hospital (protocol code: 2023-3034-Bef, date: 30.06.2023).
