# Supplementary material for: Palliative care for patients with hematologic malignancies in Germany: a nationwide survey on everyday practice and influencing factors from the perspective of treating physicians
Source: Ann Hematol. 2024 Mar 28;103(5):1753–63. doi: 10.1007/s00277-024-05726-8 (PMC11009764; doi:10.1007/s00277-024-05726-8)
Supplement: Supplementary file 1 — Supplementary Material 1: Online survey conducted and evaluated according to the CHERRIES checklist [file 277_2024_5726_MOESM1_ESM.docx]

**Supplementary Material:**
**Table S1.** Online survey conducted and evaluated according to the CHERRIES checklist

**Title: Palliative Care for Patients with Hematologic Malignancies in Germany: A Nationwide Survey on Everyday Practice and Influencing Factors from the Perspective of Treating Physicians.**

**Journal: Annals of Hematology**

**Authors:** Cordula Gebel^1+3^, Isabel Kruschel^1+3^, Steffi Bodinger^1+3^, Steffen T. Simon^2^, Dennis A. Eichenauer^4^, Anne Pralong^2^, Ulrich Wedding^1+3^

1 Department of Palliative Care, Jena University Hospital, Jena, Germany.

2 Department of Palliative Medicine, Faculty of Medicine and Cologne University Hospital, Center for Integrated Oncology Aachen Bonn Dusseldorf Cologne, Cologne, Germany

3 Comprehensive Cancer Center Central Germany (CCCG)

4 University of Cologne, First Department of Internal Medicine, Center for Integrated Oncology Aachen Bonn Dusseldorf Cologne, Cologne, Germany

**Corresponding author:** cordula.gebel@med.uni-jena.de

**Table S1.** Online survey conducted and evaluated according to the CHERRIES checklist: (Eysenbach (2004) Improving the quality of Web surveys: the Checklist for Reporting Results of Internet E-Surveys (CHERRIES). J Med Internet Res. 2004;6(3):e34-e. DOI: 10.2196/jmir.6.3.e34)

| Category | Explanation |
| --- | --- |
| Design | |
| survey design | Prospective, cross-sectional, nationwide online survey of physicians treating patients with HM. The online questionnaire was accessible through an open link. |
| IRB approval | The study was conducted in accordance with the Declaration of Helsinki and approved by the local ethics committee of the University Hospital Jena (protocol code: 2023-3034-Bef, date: 30.06.2023). This study and the APC were funded by the "Förderverein Universitätsklinikum Jena", decision date: 27.04.2023. |
| Informed consent | The survey landing page informed the participant of the background, purpose, and length of the study. It also provided contact information for the conducting institute and disclosure of potential conflicts of interest. Data handling and privacy were also explained. At the start of the survey, participants gave their consent to participate. All participants agreed that their data would be used for scientific analysis. |
| Data protection | No personal information was collected. The data generated in the course of the survey were sent in encrypted form and stored in the data center of the University Hospital Jena for an estimated period of 2-3 years until the scientific evaluation has been completed. The privacy policy of the University Hospital Jena also applies to indirectly collected and stored data, which are generated in the course of providing the web application. The privacy policy can be accessed via the following link: https://www.uniklinikum-jena.de/Datenschutz.html |
| Development and pre-testing | |
| Development and pre-testing | The development of the questionnaire was based on a comprehensive analysis of the relevant literature, comparable surveys and previously published survey instruments. A multidisciplinary review of the questionnaire was then conducted by experts (palliative care physicians, general practitioners, oncologists/hematologists and psycho-oncologists). This review covered both methodological and content aspects, assessing appropriateness, feasibility and face validity. The pretest was administered to eight physicians who treat patients with HM. This was done to ensure comprehensibility, acceptability and a balanced distribution of responses. As a result, minor adjustments were made to the questionnaire and five questions were eliminated. After these refinements, the questionnaire was considered effective and feasible. The online survey was conducted using LimeSurvey. |
| Recruitment process and description of the sample having access to the questionnaire | |
| Open survey versus closed survey | Open survey. Participants need to know the link to the survey. They will receive the link in the DGHO newsletter or by personal email. The survey is not password-protected. |
| Contact mode | Recruitment was a two-step process. First, information about the study was provided in the DGHO newsletter and participation in the online survey was requested. In the second step, individuals and practice groups identified in the database were contacted twice by personal email. A reminder was sent after two weeks. |
| Survey administration | |
| Web/E-mail | The survey was realized by LimeSurvey. |
| Context | LimeSurvey is a website for creating, storing and analyzing online surveys. The design is adapted from the Coopertate Design of the University Hospital Jena. |
| Mandatory/voluntary | Participation was voluntary. |
| Incentives | As an incentive to participate, participants had the chance to win one of ten vouchers worth €50 each. |
| Time/Date | November 10 to December 19, 2023. |
| Randomization of items or questionnaires | No. |
| Adaptive questioning | No. |
| Number of screens (pages) | 9 |
| Completeness check | All answers were voluntary. We did not check for completeness. |
| Review step | The "Back to" and "Next" buttons allowed participants to review and change their answers. Participants could save their answers to continue the survey at a later time. |
| Response rates | |
| Unique site visitor, View rate | 237 people opened the questionnaire. (response rate of 16.1%) |
| Participation | 21 people were excluded because they only completed demographic data, and another 9 people were excluded because they worked in palliative care and therefore had a potential conflict of interest. This left n =207 questionnaires for analysis (87.3% completion rate). |
| Preventing multiple entries from the same individual | |
| Cookies used | No. |
| IP check | No. Respondents' IP addresses and referrer URLs were not collected and stored. |
| Log file analysis | Participants had the option to participate multiple times, but were asked to participate only once. |
| Registration | No. |
| Analysis | |
| Handling of incomplete questionnaires | Incomplete responses are reported descriptively. |
| Questionnaires submitted with an atypical timestamp | The average time to complete the survey was recorded. The cut-off for minimum time required was 1 minute. |
| Statistical correction | No statistical correction was made. |
