# Supplementary material for: Palliative care for patients with hematologic malignancies in Germany: a nationwide survey on everyday practice and influencing factors from the perspective of treating physicians
Source: Ann Hematol. 2024 Mar 28;103(5):1753–63. doi: 10.1007/s00277-024-05726-8 (PMC11009764; doi:10.1007/s00277-024-05726-8)
Supplement: Supplementary file 2 — Supplementary Material 2: Analyzed Questions [file 277_2024_5726_MOESM2_ESM.docx]

**Supplementary Material:**
**Table S2.** Analyzed Questions

**Title: Palliative Care for Patients with Hematologic Malignancies in Germany: A Nationwide Survey on Everyday Practice and Influencing Factors from the Perspective of Treating Physicians.**

**Journal: Annals of Hematology**

**Authors:** Cordula Gebel^1+3^, Isabel Kruschel^1+3^, Steffi Bodinger^1+3^, Steffen T. Simon^2^, Dennis A. Eichenauer^4^, Anne Pralong^2^, Ulrich Wedding^1+3^

1 Department of Palliative Care, Jena University Hospital, Jena, Germany.

2 Department of Palliative Medicine, Faculty of Medicine and Cologne University Hospital, Center for Integrated Oncology Aachen Bonn Dusseldorf Cologne, Cologne, Germany

3 Comprehensive Cancer Center Central Germany (CCCG)

4 University of Cologne, First Department of Internal Medicine, Center for Integrated Oncology Aachen Bonn Dusseldorf Cologne, Cologne, Germany

**Corresponding author:** cordula.gebel@med.uni-jena.de

**Table S2.** Analyzed Questions

| Sample description |  |
| --- | --- |
| How old are you?   - Younger than 30 years - 30 to 39 years old - 40 to 49 years old - 50 to 59 years old - 60 to 69 years old - Older than 69 years |  |
| What gender do you belong to?   - female - male - diverse |  |
| Which of the following professional groups do you belong to?   - Specialist in oncology and hematology - Assistant physician - Other, namely: |  |
| How many years of experience do you have in treating hematology patients?   - less than 2 years - 2-5 years, - 5-10 years, - 11-15 years - Over 15 years |  |
| Where do you mainly work as a doctor?   - Practice - Hospital - I am not active in patient care - Other area of care: Free text |  |
| In which region is your main place of work located?   - Rural community (<5000 inhabitants) - Small town (>5000-20 000 inhabitants) - Medium-sized town (>20,000-100,000 inhabitants) - Big city (>100 000 inhabitants) |  |
| In what form did you have contact with palliative care as part of your basic, further or advanced training? (multiple answers possible)  None   - Lessons as part of medical studies/specialist training - Rotation as part of medical studies/specialist training - 40-hour course in palliative medicine (basic course) - Medical activity in a palliative care facility of at least 6 months (palliative care ward/hospice etc.) - Additional training in palliative care (“Zusatzbezeichnung Palliativmedizin”) - Approval as a doctor for particularly qualified and coordinated palliative care (“BQKPMV”) |  |
| What is the percentage of patients with hematologic malignancies among all your patients?   - <5% - 6-25% - 26-50% - 51-75% - 76-95% - >95% |  |
| How important do you personally consider palliative care for patients with hematologic malignancies to be in your own medical practice?   - A high importance - A medium importance - A peripheral importance |  |
| How common is the need for palliative medical care for patients with hematologic malignancies in your current everyday medical practice?   - Very Rarely - Rarely - Occasionally - Frequently - Very Frequently |  |
| Talking about the threat to life and the fear of dying |  |
| With what percentage of your patients with hematologic malignancies do you discuss the topic of "threat to life / fear of dying"?   - <5% - 6-25% - 26-50% - 51-75% - 76-95% - >95% |  |
| With your patients with hematologic malignancies, at what point do you typically bring up the topic of "threat to life /fear of dying"? (multiple answers possible)   - When cancer is diagnosed - At the start of treatment - During a stable phase - In case of acute deterioration - When therapy goals change - In the pre-terminal/terminal phase - Whenever patients/relatives raise the issue themselves - None of the above / unable to answer - Other, namely: Free text |  |
| Changing goals of care: Challenges and helpful resources |  |
| If, despite tumor-specific therapy, there is no longer any prospect of cure and prolonging life is not reasonable or possible, the question arises as to whether the goal of therapy should be changed. This means a change from the goal of cure to a purely supportive therapy concept (palliative or allowing the natural course of the disease) with the goal of the best possible quality of life until death. For example, it may be decided not to use intensive care measures such as mechanical ventilation or resuscitation. It may also mean stopping all life-prolonging interventions (including anti-infectives, blood substitutes).  We would like to learn more about the process of changing treatment goals in patients with malignant hematologic diseases, in particular the challenges and possible support.  From a medical perspective, what makes it challenging for you to determine when to change goals of care in patients with hematologic malignancies?   - The lack of objective parameters for the diagnosis of non-curability - Having other treatment options available, even if they are less effective - Progression with rapid deterioration of the clinical condition - Long, complicated courses after allogen Stem cell therapy (e.g. severe GvHD) with a very low chance of improvement and a high risk of death - To decide on the limitation of certain therapies (e.g. resuscitation; intensive care measures; blood transfusions; anti-infectives; immunosuppressants) - Seeing a change in goals of care as a failure of my professional role - Situations in which the prospect of improvement (and therefore the indication for the continuation of life-prolonging therapies) is assessed differently by the various professional groups or disciplines - Situations in which the patient's wishes for a change in goals of care are perceived differently by different professional groups - The patient's hope of achieving an improvement through further therapies - None of the above / unable to answer - Others, namely: Free text |  |
| Which services are or would be helpful to you in making clinical decisions about changes in goals of care and in discussing these decisions with patients and their families? (multiple answers possible)   - Have standardized clinical parameters available that indicate non-curability in the clinical course (red flags) - board on bone marrow transplantation, tumor boards and joint decision-making with other colleagues - Involving palliative care physicians in discussions with patients/families about changes in goals of care - Ethical case discussion when ethical questions or uncertainties arise in the process of changing goals of care - Training on delivering bad news (breaking bad news) - Involving psycho-oncologists in discussions with patients/families about changes in goals of care - Involving palliative care physicians in the decision-making process for changes in goals of care - Guidance in discussions by (experienced) colleagues - Observation of such discussions by (experienced) colleagues - Guidelines with key medical criteria (red flags) and support for conducting and implementing discussions - None of the above / unable to answer - Others, namely: |  |
| Factors influencing PC-integration |  |
| ADDRESSING PALLIATIVE CARE NEEDS MYSELF  The following are statements about the palliative medical treatment of your patients with hematologic malignancies as part of their medical care.  To what extent do you agree with the following statements? |  |
| \| The lack of time hinders the treatment of my patients' palliative needs. \| - Do not agree at all - Do not agree - I neither agree nor disagree - Agree - Fully Agree \| \| --- \| --- \| \| I am able to treat the physical symptoms of my patients myself as part of the palliative care (e.g., pain, shortness of breath). \| - Do not agree at all - Do not agree - I neither agree nor disagree - Agree - Fully Agree \| \| I am able to treat the psychological problems (e.g. anxiety, depression) of my patients myself as part of the palliative care. \| - Do not agree at all - Do not agree - I neither agree nor disagree - Agree - Fully Agree \| \| I feel competent when it comes to questions of spirituality in the context of palliative care (for example, the meaning of life). \| - Do not agree at all - Do not agree - I neither agree nor disagree - Agree - Fully Agree \| \| I feel competent to plan care/resolve social problems related to palliative care (e.g., living alone). \| - Do not agree at all - Do not agree - I neither agree nor disagree - Agree - Fully Agree \| \| Dealing with the palliative needs of my patients is challenging for me because of the emotional impact. \| - Do not agree at all - Do not agree - I neither agree nor disagree - Agree - Fully Agree \| \| The palliative needs of patients are routinely addressed by my specialist colleagues (in the clinic/outpatient clinic). \| - Do not agree at all - Do not agree - I neither agree nor disagree - Agree - Fully Agree \| \| Other challenges and solutions in addressing patients' palliative care needs are: \|  \| | |

| REFERRING PATIENTS TO PALLIATIVE CARE  The referral of patients with hematological malignancies to specialized palliative care teams (SPHC, hospice, hospital palliative care service, palliative care unit) is a challenge... | | |
| --- | --- | --- |
|  | ... because there are too few specialized palliative care providers available in my region. | - Do not agree at all - Do not agree - I neither agree nor disagree - Agree - Fully Agree |
|  | ... because too much time passes between the referral and the care provided by the specialized palliative care teams. | - Do not agree at all - Do not agree - I neither agree nor disagree - Agree - Fully Agree |
|  | ... because the criteria of the specialized palliative care providers are too restrictive to meet the needs of my patients (e.g. exclusion of patients with blood replacement procedures) | - Do not agree at all - Do not agree - I neither agree nor disagree - Agree - Fully Agree |
|  | ... because I don't know which specialized palliative care providers are available in my region. | - Do not agree at all - Do not agree - I neither agree nor disagree - Agree - Fully Agree |
|  | ... because the specialized palliative care providers in my region do not have sufficient knowledge and experience with hematological malignancies . | - Do not agree at all - Do not agree - I neither agree nor disagree - Agree - Fully Agree |
|  | ... because patients have negative perceptions of "palliative care". | - Do not agree at all - Do not agree - I neither agree nor disagree - Agree - Fully Agree |
|  | ... because it is difficult to find the right time for a referral. | - Do not agree at all - Do not agree - I neither agree nor disagree - Agree - Fully Agree |
|  | ... because it is unclear how patients can be referred to specialized palliative care services. (Referral process) | - Do not agree at all - Do not agree - I neither agree nor disagree - Agree - Fully Agree |
|  | Are there other challenges in referring your patients to specialized palliative care? |  |
| WORKING WITH PALLIATIVE CARE TEAMS  WORKING with specialized palliative care teams (SPHC, hospice, hospital palliative care service, palliative care unit) is a challenge... | | |
|  | ... because of role confusion when multiple professionals are involved (i.e., who does what). | - Do not agree at all - Do not agree - I neither agree nor disagree - Agree - Fully Agree |
|  | ... because of the lack of a standard process for professional communication between teams (e.g., knowing who, when, and how to contact each other; access to home care records). | - Do not agree at all - Do not agree - I neither agree nor disagree - Agree - Fully Agree |
|  | ... because of the lack of standard processes for executing new orders for patients who are at home(e.g. ordering a puncture). | - Do not agree at all - Do not agree - I neither agree nor disagree - Agree - Fully Agree |
|  | Are there other challenges in working with specialized palliative care services? |  |

Note: The English translations of the original German questions are presented. SPHC=Specialist palliative home care.
