# Supplementary material for: Palliative care for patients with hematologic malignancies in Germany: a nationwide survey on everyday practice and influencing factors from the perspective of treating physicians
Source: Ann Hematol. 2024 Mar 28;103(5):1753–63. doi: 10.1007/s00277-024-05726-8 (PMC11009764; doi:10.1007/s00277-024-05726-8)
Supplement: Supplementary file 3 — Supplementary Material 3: Themes from qualitative data [file 277_2024_5726_MOESM3_ESM.docx]

**Supplementary Material:**
**Table S3**. Themes from qualitative data

**Title: Palliative Care for Patients with Hematologic Malignancies in Germany: A Nationwide Survey on Everyday Practice and Influencing Factors from the Perspective of Treating Physicians.**

**Journal: Annals of Hematology**

**Authors:** Cordula Gebel^1+3^, Isabel Kruschel^1+3^, Steffi Bodinger^1+3^, Steffen T. Simon^2^, Dennis A. Eichenauer^4^, Anne Pralong^2^, Ulrich Wedding^1+3^

1 Department of Palliative Care, Jena University Hospital, Jena, Germany.

2 Department of Palliative Medicine, Faculty of Medicine and Cologne University Hospital, Center for Integrated Oncology Aachen Bonn Dusseldorf Cologne, Cologne, Germany

3 Comprehensive Cancer Center Central Germany (CCCG)

4 University of Cologne, First Department of Internal Medicine, Center for Integrated Oncology Aachen Bonn Dusseldorf Cologne, Cologne, Germany

**Corresponding author:** cordula.gebel@med.uni-jena.de

**Table S3**. Themes from qualitative data

|  | Themes | Subtheme | Exemplar quote |
| --- | --- | --- | --- |
|  | Talking about the threat to life and the fear of dying | | |
|  | Time | Depending on entity / prognosis | "It depends on the type of hematologic disease. For MDS-EB or AML at diagnosis, for CLL only at deterioration or terminal phase" (ID 136, Specialist in oncology and hematology, Practice) |
|  |  | Depending on patients | "Patients signal very clearly if they don't want to talk about the issue, I respect that kind of repression, otherwise I try to address it whenever it's appropriate in the relationship with the patient and the stage of their individual processing."  (ID170, Specialist in oncology and hematology, Practice) |
|  |  | Trigger | "before SCT" (ID59, Specialist in oncology and hematology, Hospital) |
|  | Type and manner of the conversation | Early & open | "I address the topic openly as early as possible (also in detail), especially in acute cases (e.g. high-risk MDS, AML)." (ID10,  Specialist in oncology and hematology, Hospital) |
|  |  | Depending on the situation | "Addressing the topic with the patient is more often situational" (ID131, Specialist in oncology and hematology, Hospital) |
|  |  | Repeated | "In my view, the topic should be addressed repeatedly whenever there is a need from a physician's or patient's perspective." (ID137, Specialist in oncology and hematology, Hospital) |
|  |  | Integration of theme | "I would not separate these topics of conversation from other topics of conversation, because in the case of hematological malignancies (however defined), this topic is always present." (ID19), Specialist in oncology and hematology, Hospital) |
|  |  | |  |
|  | Lack of Time | | "Conversations about life-threatening situations and fear of dying are part of every discussion with patients and relatives, but unfortunately - like all conversations - there is too little space/time for them," (ID194, Specialist in oncology and hematology, Hospital) |
|  | Change in goals of care | | |
|  | Challenges | Different perspectives patient and family | "Situations in which relatives cannot accept a change in treatment goals and the patient cannot tolerate/resolve the conflict.“ (ID170, Specialist in oncology and hematology, Practice) |
|  |  | Rejection by the patient | "Patients don't want to talk about it" (ID161, Specialist in oncology and hematology, Hospital) |
|  |  | Specific patient group | "Young patients" (ID233, Assistant physician, Hospital) |
|  |  | Burden of therapy goal changes for physicians and families | "The decision to stop anti-infectives (...) and transfusions (...) in particular is often very difficult for physicians, relatives and nursing staff" (ID16, Specialist in oncology and hematology, Hospital) |
|  |  | New treatment options | "The variety of treatment and study options in particular often means that patients with a poor prognosis are often given hope (even by senior physicians). As a result, the palliative care connection is delayed." (ID191, Assistant physician, Hospital) |
|  |  | Established culture | "The predominantly poor palliative medical care of hematology patients is often caused by the hematologists treating them. Observing these conversations for young colleagues is therefore not helpful either. " (ID40, Specialist in oncology and hematology, Hospital) |
|  |  |  |  |
|  | Helpful resources | Definitions | "In my opinion, a clear differentiation between the end of anti-tumor therapy and the end of anti-infectives/transfusions is necessary." (ID16, Specialist in oncology and hematology, Hospital) |
|  |  | Integration into medical studies | "It's better to learn to "talk to patients" right from the start (during medical studies) and "practise" in everyday life, but don't just skip/skip out and also have the courage to make mistakes (breaking bad news can sometimes go badly)." (ID87, Specialist in oncology and hematology, Practice) |
|  |  | Other actors | "Translator / Cultural Consultant” (ID94, Specialist in oncology and hematology, Hospital) |
|  | Collegial sharing and team consultations | Challenge | "In fact, I don't always find the tumor conferences helpful, because treatment options are often discussed there that are conceivable according to the guidelines, but are not feasible in reality." (ID146, Specialist in oncology and hematology, Hospital) |
|  |  | Resource | "In my opinion, the most important thing is to discuss the case together with all the professional groups involved and decide on the change of treatment goal. Only if everyone is behind the decision will it be jointly represented to the patient." (ID177, Specialist in oncology and hematology, Hospital) |
|  | Responsibility of the physician | | "I don't think it's good if the discussions are "delegated" to other professionals - the change in treatment goal should be discussed by the physician who is in charge. Assistant physicians, for example, think that palliative care units are great because they do not have to have the difficult conversations themselves." (ID170, Specialist in oncology and hematology, Practice) |
|  | Factors influencing palliative care integration | | |
|  | General conditions | Good clinic structure/  networks | "There is good cooperation with psycho-oncologists and cancer counseling services in the city" (ID228, Specialist in oncology and hematology, Practice) |
|  |  | Not enough capacity | "The palliative care team in our area is good, but there are not enough places, especially hospices have long waiting times." (ID233, Assistant physician, Hospital) |
|  | Barriers | Lack of knowledge about hematologic diagnosis and progression among palliative care providers | "Very often high level of ignorance regarding hematological diagnoses and corresponding complications (infections, transfusions). Very often readmissions despite SPHC referral (often also due to ignorance) Often unsettled patients, as SPHC services make ignorance clear." (ID16, Specialist in oncology and hematology, Hospital) |
|  |  | Restrictive conditions imposed by palliative care providers | "One difficulty with the connection is that the SPHC teams and hospices give us strict deadlines as to when the patient has to be at home and when the medication plan has to be faxed. Otherwise, we can only discharge the patient on another day." (ID205, Specialist in oncology and hematology, Hospital) |
|  | Attitude of the physician | Integration palliative care | "In my personal experience: Palliative medicine should only begin when all oncological options have been exhausted or are not desired." (ID23, Specialist in oncology and hematology, Practice) |
|  |  | Talking about | "There is a large difference, even within the professional group, depending on the physician's personal view of when to talk about limiting treatment" (ID77, Assistant physician, Hospital) |
|  | Departmental Responsibilities | | "In the case of medical problems, therapy is provided by myself/your own department or, if necessary, by the specialist department; for non-medical issues, referral/presentation is made to the relevant departments (social services/psychoncology/hospital chaplain etc.)." (ID185, Specialist in oncology and hematology, Hospital) |
|  | Positive experiences | | "Neither referral nor cooperation is a challenge in our region" (ID78, Specialist in oncology and hematology, Hospital) |

Note: The English translations of the original German quotes are presented. SPHC=Specialist palliative home care
